# Propionic acid supplementation promotes the expansion of regulatory T cells in patients with end-stage renal disease but not in renal transplant patients

**DOI:** 10.3389/frtra.2024.1404740

**Published:** 2024-09-09

**Authors:** Moritz Anft, Fabian Meyer, Sirin Czygan, Felix S. Seibert, Benjamin J. Rohn, Fotios Tsimas, Richard Viebahn, Timm H. Westhoff, Ulrik Stervbo, Nina Babel, Panagiota Zgoura

**Affiliations:** ^1^ Center for Translational Medicine and Immune Diagnostics Laboratory, Medical Department I, Marien Hospital Herne, University Hospital of the Ruhr-University Bochum, Herne, Germany; ^2^ Department of Anesthesiology, Knappschaftskrankenhaus Bochum, Bochum, Germany; ^3^ Department of Surgery, Knappschaftskrankenhaus Bochum, Bochum, Germany; ^4^ Berlin Institute of Health, Berlin-Brandenburg Center for Regenerative Therapies, and Institute of Medical Immunology, Charité—Universitätsmedizin Berlin, Corporate Member of Freie Universität Berlin, Humboldt-Universität zu Berlin, Berlin, Germany; ^5^ Clinic for Internal Medicine, St. Anna Hospital Herne, Herne, Germany

**Keywords:** immunity, immunosuppression, kidney transplantation, propionate, regulatory T cells

## Abstract

In a previous study, we showed an anti-inflammatory effect of propionic acid supplementation in dialysis patients. The present study intends to analyze the effect of propionic acid on the chronic inflammatory state and T-cell composition in kidney transplant patients compared to dialysis patients. A total of 10 dialysis patients and 16 kidney transplant patients under immunosuppressive standard triple immunosuppressive therapy received 2 × 500 mg propionic acid per day for 30 days. The cellular immune system was analyzed before and after the propionic acid supplementation and 30–90 days thereafter as a follow-up. We measured the main immune cell types and performed an in-depth characterization of T cells including regulatory T cells (Tregs), B cells, and dendritic cells. In addition, we assessed the functional activity and antigenic responsiveness by analysis of third-party antigen-specific T cells after their stimulation by recall (tetanus diphtheria vaccine) antigen. In dialysis patients, we observed an expansion of CD25^high^CD127^−^ Tregs after propionic acid intake. In contrast, the same supplementation did not result in any expansion of Tregs in transplant patients under immunosuppressive therapy. We also did not observe any changes in the frequencies of the main immune cell subsets except for CD4^+^/CD8^+^ distribution with an increase of CD4^+^ T cells and decrease of CD8^+^ T cells in the transplant population. Our data suggest that dietary supplements containing propionate might have a beneficial effect decreasing systemic inflammation in dialysis patients through Treg expansion. However, this effect was not observed in transplant patients, which could be explained by counteracting effect of immunosuppressive drugs preventing Treg expansion.

## Introduction

Patients with end-stage renal disease (ESRD) requiring kidney replacement treatment by either hemodialysis or kidney transplant face an imbalance of the immune system for various reasons. On the one hand, ESRD patients experience chronic inflammatory conditions due to oxidative stress, intestinal dysbiosis, decreased cytokine elimination, or frequent infections, which are associated with cardiovascular events, the primary cause of increased mortality and morbidity (1–3). On the other hand, ischemia/reperfusion injury in the context of transplant surgery and alloimmune processes occurring because of permanent stimulation of recipient immunity by donor human leukocyte antigen (HLA) molecules after kidney transplantation lead to an increased inflammatory stage in the transplant patient (4). Therefore, both patient groups benefit from a natural reduction of pro-inflammatory conditions in the body.

Propionic acid is a naturally occurring fatty acid produced during the fermentation of dietary fibers by specific gut bacteria. It is found in various food sources, including dairy products, certain types of cheese, and some grains, and is the main component of short-chained fatty acids (SCFA) (5–7). In recent years, scientific interest in propionic acid has intensified as evidence suggests its involvement in modulating immune responses, reducing inflammation, and overall immune system homeostasis. It was shown that in the blood of humans who were supplemented with sodium propionate, the pro-inflammatory factors CRP, IL-2, and IL-17 were significantly reduced and the anti-inflammatory factors TGF-β and IL-10 increased (8). This ability to modulate cytokine production suggests its potential in maintaining immune homeostasis.

In addition, a supplementation with propionic acid was associated with the expansion of regulatory T cells (Tregs), which play crucial role in preventing excessive immune responses and maintaining immune tolerance (9–11). In our previous study on hemodialysis patients as well as in another study on multiple sclerosis patients, the frequencies of CD25^high^CD127^−^ Tregs were significantly increased after supplementation with propionic acid for 30 days (10, 12). Propionic acid may contribute to inducing mechanisms of immune regulation and thereby support the body's efforts to combat persistent chronic inflammation or autoinflammatory diseases.

A triple immunosuppressive therapy consisting of calcineurin inhibitors (CNI), mycophenolate mofetil (MMF), and steroids is used for effective suppression of the immune system in patients undergoing renal transplantation. CNIs selectively inhibit T cells by blocking the serine–threonine phosphatase calcineurin, thereby inhibiting the transcription factor NFAT and suppressing cell activation and pro-inflammatory cytokine production (13). In contrast, MMF selectively inhibits *de novo* proliferation of B and T cells by blocking inosine monophosphate dehydrogenase (IMPDH) (14). The net effect of these immunosuppressants determines the overall degree of the immune suppression and its tolerance toward transplanted organ as well as protective function against infection with bacteria/viruses. The aim of this study was to investigate whether kidney transplant patients on triple immunosuppressive therapy benefit from propionic acid supplementation and whether there is an expansion of regulatory T cells comparable to hemodialysis patients.

## Results

### Analysis of regulatory T cells

**Figure 1:**
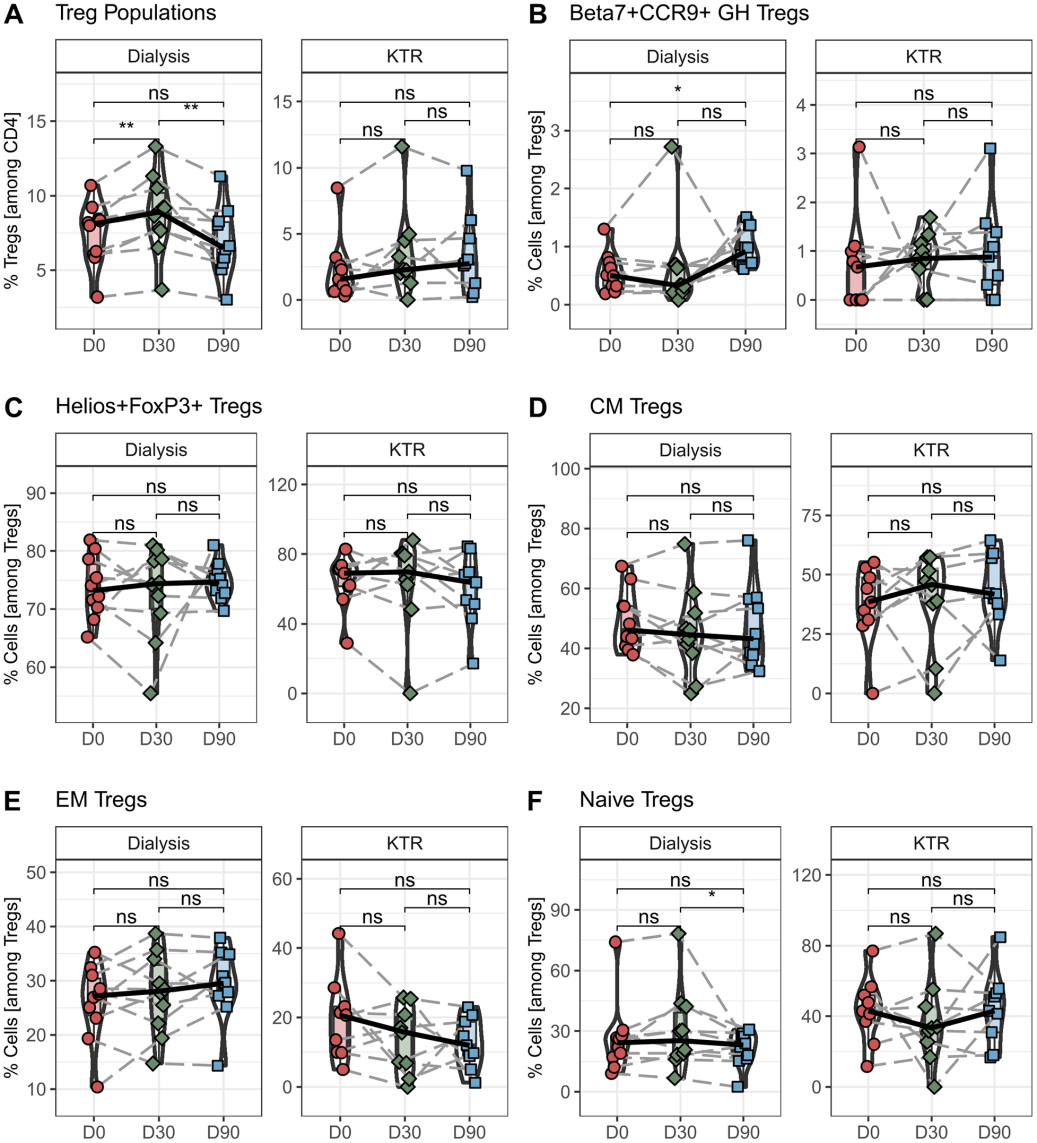
Percentage of regulatory T cells (Tregs) in hemodialysis patients and kidney transplant recipients (KTR) after propionic acid treatment. Peripheral blood mononuclear cells from hemodialysis patients and KTR were isolated at the beginning (D0) and end (D30) of propionic acid supplementation and 60 days thereafter (D90), stained with the appropriate antibodies and analyzed in a flow cytometer. **(A)** Regulatory T cells were identified as CD3+CD4+CD25+FoxP3+. **(B,C)** In the Treg population, gut homing (GH) Tregs were identified as Beta7+CCR9+ and thymus generated natural Tregs (nTregs) as Helios+. **(D–F)** Central memory (CM), effector memory (EM), and naïve T cells were identified by CCR7 and CD45RA. Pairwise Mann–Whitney *U* test was performed. ns, not significant. *p-value < 0.05.

The aim of the study was to investigate the effect of propionic acid supplementation on patients after kidney transplantation and to compare these results with hemodialysis patients. In the first step, we analyzed the percentage of regulatory T-cell populations before and after propionic acid supplementation. In transplant patients, we did not observe an increase in the frequency of FoxP3^+^ or Helios^+^FoxP3^+^ regulatory T cells after propionic acid intake (Figure 1A). In contrast, we could show an increase of regulatory T cells during propionic acid intake in hemodialysis patients (Figure 1A). Furthermore, we detected a gut-homing Beta7^+^CCR9^+^ Tregs expansion in hemodialysis patients up to 90 days after the start of propionic acid supplementation (Figure 1B). In contrast, kidney transplant patients under immunosuppression showed no expansion of gut-homing Beta7^+^CCR9^+^ Tregs 30 days after propionic acid intake. When examining the memory cell distribution before and after the administration of propionic acid, we could not find any changes in the frequency of Helios^+^FoxP3^+^, central memory, and effector memory or naïve T cells within the transplant patient population (Figures 1C–F).

The present study shows that in unlike to hemodialysis patients, kidney transplant patients do not experience an expansion of regulatory T cells after propionate supplementation.

### Analysis of the main immune cell populations

**Figure 2:**
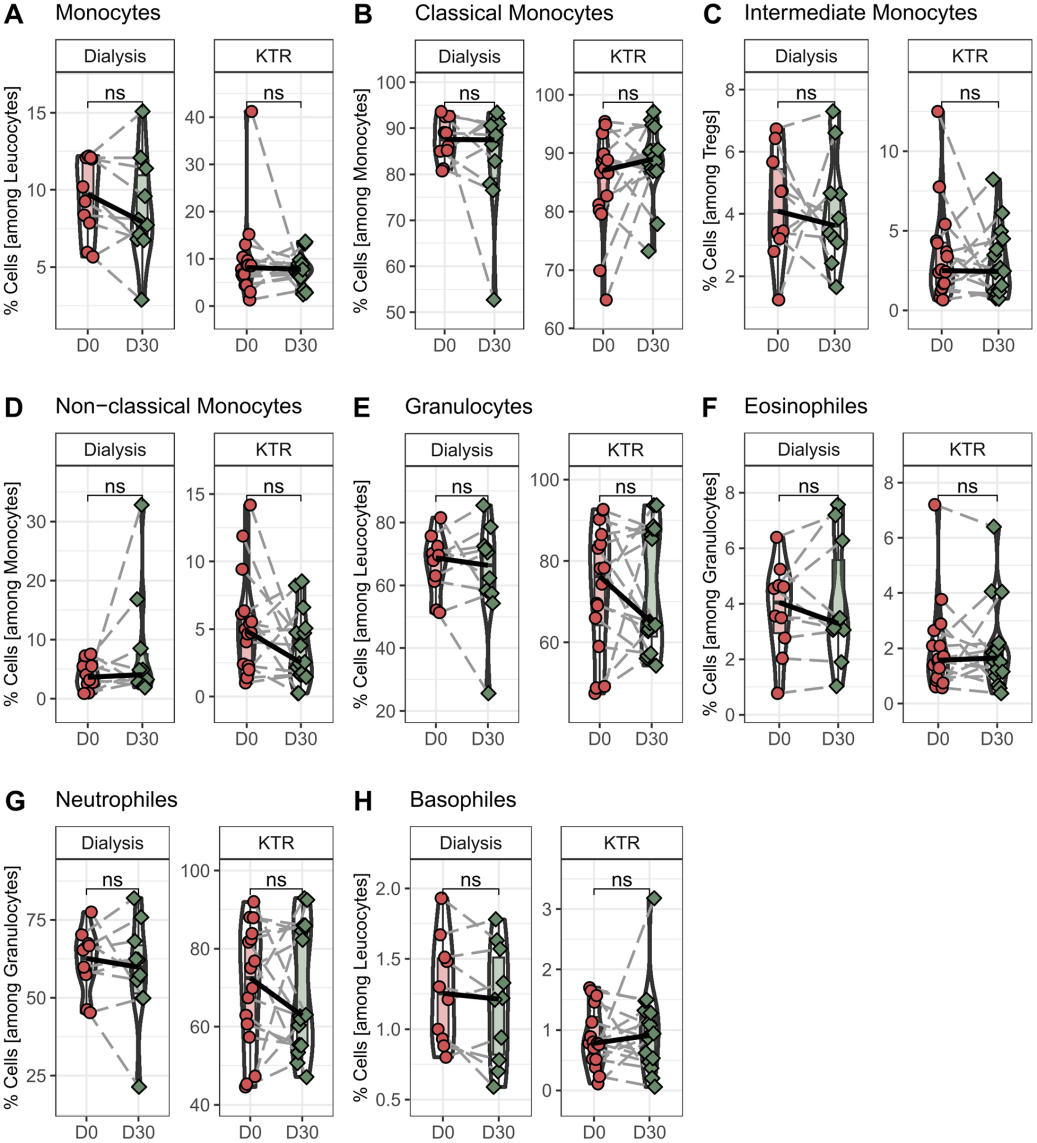
Percentage of monocyte and granulocyte populations in hemodialysis patients and kidney transplant recipients (KTR) before and after propionic acid treatment. Peripheral blood mononuclear cells from hemodialysis patients and KTR were isolated at the beginning (D0) and end (D30) of propionic acid supplementation stained with the appropriate antibodies and analyzed in a flow cytometer. **(A–D)** Monocytes were identified by sidescatter profile and CD45, CD14, and CD16 expression. **(E–H)** Granulocytes were identified by sidescatter profile, CD45, and CD16 expression. Pairwise Mann–Whitney *U* test was performed. ns, not significant. *p-value < 0.05.

To further analyze the effect of propionic acid intake on other immune cells, we performed an in-depth analysis of the cellular immune system. Due to the lack of samples, only the time before and after propionate administration (D0 + D30) could be compared in the following analyses. At first, we looked into the monocyte and granulocyte populations. We found no differences in the general CD14^+^ monocytes in kidney transplant patients after propionic acid supplementation and no alterations in the CD14^+^CD16^−^ classical monocytes, CD14^+^CD16^+^ intermediate monocytes, or CD14^dim^CD16^+^ non-classical monocytes subpopulations (Figures 2A–D). We also found no differences in either general granulocytes or in CD16^+^ neutrophils, CD16^−^ eosinophils, or basophiles in transplant patients under immune suppression after propionic acid supplementation (Figures 2E–H). These results are consistent with the analysis of monocyte and granulocyte populations after propionic acid administration in hemodialysis patients, showing no changes after 30 days of supplementation.

**Figure 3:**
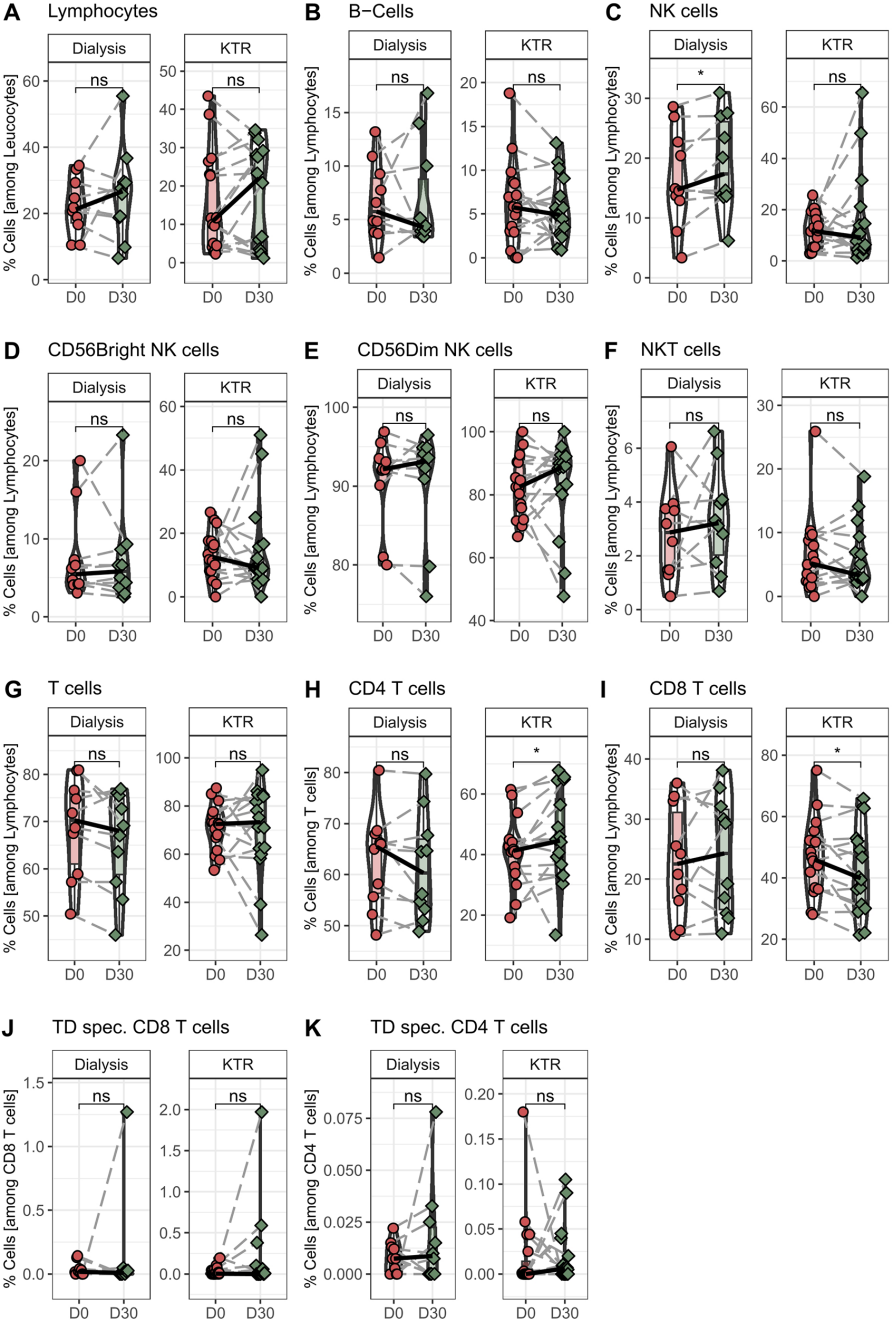
Percentage of monocyte and granulocyte populations in hemodialysis patients and kidney transplant recipients (KTR) before and after propionic acid treatment. PBMCs from hemodialysis patients and KTR were isolated at the beginning (D0) and end (D30) of propionic acid supplementation stained with the appropriate antibodies and analyzed in a flow cytometer. **(A–I)** Lymphocytes were identified by sidescatter profile and CD45. B cells as CD19+CD3-, natural killer (NK) cells as CD3-CD56+, natural killer T (NKT) cells as CD3+CD56+, and T cells as CD3+ and CD4+ or CD8+. **(J,K)** Peripheral blood mononuclear cells were stimulate for 18h with tetanus-diphtheria (TD) vaccine and stained with the appropriate antibodies and analyzed in a flow cytometer. Activated T helper cells were identified as CD4+ CD154+CD137+ and activated cytotoxic T cells as CD8+CD137+. Pairwise Mann–Whitney *U* test was performed. ns, not significant. *p-value < 0.05.

In the next step, we wanted to determine the effect on lymphocytes and the main lymphocyte subpopulations after 30 days of propionic acid supplementation. We found no differences in total lymphocyte percentage after 30 days of propionic acid administration in transplant patients, similar to the data on hemodialysis patients (Figure 3A). In addition, we did not detect any changes in B, NKT, and NK cells in transplant patients (Figures 3B–F), which was also consistent with our results of propionic acid supplementation in hemodialysis patients. Interestingly, we found a subpopulation shift within CD3^+^ T cells in transplant patients. Although total T cells among lymphocytes did not change after 30 days of propionic acid administration, the percentage of CD4^+^ T cells significantly increased and CD8^+^ T cells were significantly decreased (Figures 3G–I).

### Analysis of functionality of conventional T cells toward third-party antigens

In the last step, we investigated the extent to which T cells were able to respond specifically to a stimulus and become activated after 30 days of propionic acid stimulation in transplant patients. For this purpose, isolated peripheral blood mononuclear cells (PBMCs) of the patients were stimulated with tetanus diphtheria vaccine (TD) as the recall antigen for 18 h. Subsequently, activated CD154^+^CD137^+^ CD4^+^ T-helper cells and CD137^+^ CD8^+^ cytotoxic T cells were quantified. We could not find any alterations in the magnitude of TD-specific CD4^+^ and CD8^+^ T cells after propionic acid administration in transplant patients as well as in hemodialysis patients (Figures 3J, K).

In conclusion, no significant differences in the composition of the cellular immune system were observed in kidney transplant recipients after propionate supplementation (Supplementary Figure S1).

## Discussion

Kidney transplant patients require permanent immunosuppression to prevent rejection of the transplant by the cellular immune system. This maintenance immunosuppressive therapy consisting usually of calcineurin inhibitors, antimetabolites, and corticosteroids must be balanced to prevent the body from being attacked by pathogens or the development of cancer diseases. Supporting immunosuppressive therapies with less drastic medications, which strengthen anti-inflammatory and immunoregulatory functions in the body, makes the study of short-chain fatty acids interesting. In addition, the long-term treatment with immunosuppressants is associated with numerous side effects such as nephrotoxicity and vascular diseases, which makes a reduction in immunosuppressant medication desirable (15, 16). In this context, propionic acid supplementation has already been shown to have a stimulatory effect on Tregs in hemodialysis and multiple sclerosis (MS) patients (10, 12). Data on adoptive transfer of autologous regulatory T cells supporting and partially replacing drug-induced immunosuppression by inhibiting conventional T cells demonstrates the importance of Tregs in transplant setting (17, 18). In this study we showed that supplementation of 2 × 500 mg propionic acid for 30 days in kidney transplant patients does not lead to an expansion of CD25^high^CD127^−^ Tregs, nor significant other changes in the distribution of the cellular immune system, as was shown in hemodialysis patients.

The exact effect of the expansion of Tregs in hemodialysis patients as well as in healthy individuals through supplementation with propionic acid is not yet fully understood. However, the fact that we could not observe an increase in regulatory T cells in kidney transplant patients after supplementation with propionic acid in contrast to hemodialysis patients is most likely attributable to the immunosuppression applied in transplant patients. Most patients received triple immunosuppression with tacrolimus, mycophenolate mofetil, and glucocorticoids. These immunosuppressants primarily act on lymphocytes and especially on T cells. For instance, tacrolimus inhibits the calcineurin-NFAT axis, thus preventing IL-2 transcription, resulting in suppression of T cells including Tregs (19, 20). Although the patients received a rather low dose of immunosuppression due to their average transplant age of more than 38 months, it is likely that this T-cell inhibition is already strong enough to prevent any stimulating effect of propionic acid on Tregs.

The effect of propionic acid on cellular immunity in hemodialysis patients was very short-lived and reversible after stopping supplementation. Interestingly, a significant increase in the percentage of gut-homing Tregs was observed only after 90 days, not after 30 days. It is not clear how this effect occurs, but it may be because the sustained anti-inflammatory effect of propionate may promote the recruitment and retention of Tregs in the gut environment (12). It is likely that 30 days of supplementation in severely immunosuppressed kidney transplant patients is not sufficient to achieve this form of propionate-induced effect at the cellular level. Since the mechanism of action of propionic acid on the cellular immune system has not yet been clarified, there is still no experience of how long it requires for propionic acid to provide a stimulating effect on regulatory T cells under different conditions. Although 30 and 14 days of supplementation with 500 mg propionic acid in hemodialysis patients (12) and MS patients (10), respectively, were sufficient to observe an expansion of Tregs, it cannot be ruled out that a longer period of time is required for this in patients under long-term immunosuppression. In addition, *in vitro* data show that the immunomodulating effect of short-chain propionic acid is dose-dependent. PBMCs supplemented with different doses of propionic acid and stimulated with lipopolysaccharides (LPS) show significant less IL-1β and TNF-α secretion when given 20 or 200 µM propionic acid compared to 2 µm (11). It is possible that in immunosuppressed kidney transplant patients a higher dose is necessary to observe a significant effect than the dose in ESRD patients.

Interestingly, we saw no differences in the percentage of CD3^+^ but a significant shift in the ratio of CD3^+^CD8^+^ to CD3^+^CD4^+^ in kidney transplant patients after 30 days of propionic acid supplementation. Whether this shift is due to an increase or decrease in a specific T-helper or cytotoxic T subpopulations could not be conclusively clarified in this study. Although a decrease in T helper 17 cells has been demonstrated in mice models (9) and humans (10) after propionic acid supplementation, no such data are known for CD8T cells.

The limitations of the present study are the small number of patients, a monocentric study design, and the lack of a microbiome analysis, which could have clarified the effect of propionic acid on the gut microbiome of the patients. In addition, the transplant patients were, on average, younger than the ESRD patients, which may have an impact on the functionality of the immune system (21). Nevertheless, this study shows that supplementation with propionic acid does not lead to a supportive expansion of Tregs in patients undergoing immunosuppression after transplantation, as is the case in hemodialysis patients. Further studies could evaluate clinical and immunological long-term effects. In this context, higher doses might be considered to achieve the immunomodulatory effect observed in patients without immunosuppressive therapy.

## Methods

### Study design and patient characteristics

In this prospective study, 16 patients with kidney transplantation and under immunosuppression were enrolled and supplemented with 2 × 500 mg propionic acid per day for 30 days. Blood was drawn and analyzed on days 0 and 30 of propionic acid supplementation and 60 days after the last administration. The exclusion criteria for ESRD and transplant patients were (1) medication with antibiotics within the last 4 weeks; (2) malignancies currently or in the past; (3) serious intestinal diseases that cause chronic diarrhea; (4) lack of informed consent; and additionally for transplant patients (5) fundamental changes in immunosuppression; and (6) acute transplant rejection. The isolated PBMCs were used for in-depth characterization of the immune cell populations by multicolor flow cytometry. These results were compared with those of ESRD patients, also supplemented with propionic acid for 30 days in a previous study (12). Demographics and clinical characteristics of patients are shown in Table 1. The immunosuppression for day 0 is shown in Table 1 and was not changed during the study. The mean age of the kidney transplant patients was 51 years and that of the hemodialysis patients 73 years. The study population was tested for age-related differences and no differences were found. Of the 16 kidney transplant patients, 13 were examined, on average, 20 months after transplantation and received triple immunosuppression with tacrolimus, glucocorticoids, and MMF or azathioprine. Of these patients, three received either everolimus, belatacept, or cyclosporine A instead of tacrolimus. Three further patients were analyzed, on average, 120 months after transplantation and received dual immunosuppression with tacrolimus and glucocorticoids (Table 1).

**Table 1:** Cohort characteristics.

	Kidney transplant patients	Hemodialysis patients
Number	16	10
Age (mean ± SD)	51.9 ± 8.3	73.8 ± 10.2
Sex [female, *n* (%)]	8 (50%)	2 (20.0%)
Months of hemodialysis (mean ± SD)	—	66.7 ± 81.6
Months of transplantation (mean ± SD)	38.8 ± 44.3	—
Transplant type [deceased donor, *n* (%)]	16 (100%)	—
Immunosuppression		
Glucocorticoids {n [dd (IQR)]}	16 [5 (5–5) mg]	0
Tacrolimus {n [dd (IQR)]}	13 [3 (2–4.5) mg]	0
MPA {n [dd (IQR)]}	9 [1,440 (875–2,000) mg]	0
Azathioprine {n [dd (IQR)]}	75 [3 (50–100) mg]	0
Cyclosporin A [n (dd)]	1 (300 mg)	0
Belatacept [n (dd)]	1 (5 mg/kg BW)	0
Everolimus [n (dd)]	1 (3 mg)	0

n, number; SD, standard deviation; dd, median daily dose; IQR, interquartile range; BW, body weight; MPA, mycophenolic acid.

### Preparation of PBMCs

Peripheral blood was collected in S-Monovette K3 ethylenediaminetetraacetic acid (EDTA) blood collection tubes (Sarstedt, Nümbrecht, Germany). Collected blood was pre-diluted in phosphate-buffered saline (PBS)/bovine serum albumin (BSA) (Gibco Thermo Fisher Scientific, Waltham, USA) at a 1:1 ratio and underlaid with 15 ml Ficoll-Paque Plus (GE Healthcare, Chicago, USA). Tubes were centrifuged at 800 g for 20 min at room temperature. Isolated PBMCs were washed twice with PBS/BSA and stored at −80°C until use as previously described (22).

### T-cell stimulation assay

Isolated PBMCs were stimulated with 10 µl tetanus-diphtheria-adsorbate vaccine [TD-pur, Sanofi Pasteur MSD, France; Tetanus-Toxoid ≥ 40 international unit (IU); diphtheria-Toxoid ≥ 4 IU/ml]. A total of 2.5 × 10^6^ PBMCs were plated for each condition in 96-UWell Plates in RPMI media (Life Technologies, Carlsbad, USA), supplemented with 1% Penicillin-Streptomycin-Glutamine (Sigma Aldrich, St. Louis, USA), and 10% FCS (PAN Biotech, Aidenbach, Germany), and were stimulated or left untreated as a control for 16 h. As a positive control, cells were stimulated with SEB (1 µg/ml; Sigma Aldrich) or left untreated as a negative control. After 2 h, Brefeldin A (1 µg/ml; Sigma Aldrich) was added. As previously applied by our groups and others, antigen-specific responses were considered positive after the non-specific background was subtracted, and more than 0.001% or at least 15 positive cells were detectable. Negative values were set to zero.

### Flow cytometry

EDTA-treated whole blood was stained with optimal concentrations of each antibody for 10 min at room temperature in the dark. Erythrocytes were lysed using VersaLyse (Beckman Coulter, Brea, USA) with 2.5% IOTest 3 Fixative Solution (Beckman Coulter, Brea, USA) for 30 min at room temperature in the dark. The gating strategy is shown in Supplementary Figure S2.

Panels for Tregs and stimulated T cells were extracellular and stained with optimal concentrations of antibodies for 10 min at room temperature in the dark. Cells were washed twice with PBS/BSA before preparation for intracellular staining using the Intracellular Fixation and Permeabilization Buffer Set (Thermo Fisher Scientific, Waltham, USA) as per the manufacturer's instructions. Fixed and permeabilized cells were stained for 30 min at room temperature in the dark with an optimal dilution of antibodies against the intracellular antigen. Gating strategies for Tregs and TD-specific T cells are shown in Supplementary Figures S3 and S4.

All samples were immediately acquired on a CytoFlex flow cytometer (Beckman Coulter). Quality control was performed daily using the recommended CytoFlex Daily QC Fluorospheres (Beckman Coulter). No modification to the compensation matrices was required throughout the study. Flow cytometry data were analyzed using FlowJo version 10.6.2 (BD Bioscience, Franklin Lakes, USA).

### Statistical analysis

Statistical analysis was performed using R (R Core Team), version 4.2.1. Categorical variables are summarized as numbers and frequencies; quantitative variables are reported as median and interquartile range. Box plots and violin plots depict the median and the first and third quartiles. The whiskers correspond to 1.5 times the interquartile range. All applied statistical tests are paired and two-sided. Differences in quantitative variables between all three groups are analyzed using the non-parametric Wilcoxon test. *p*-values <0.050 were considered significant. *p*-values were not corrected for multiple testing, as this study was of exploratory nature.

### Study approval

The study was approved by the ethical committee of the Ruhr University Bochum (20-6886). Written informed consent was obtained from all participants.

## Data Availability

The raw data supporting the conclusions of this article will be made available by the authors, without undue reservation.
