# Supplementary material for: Propionic acid supplementation promotes the expansion of regulatory T cells in patients with end-stage renal disease but not in renal transplant patients
Source: Front Transplant. 2024 Sep 9;3:1404740. doi: 10.3389/frtra.2024.1404740 (PMC11425579; doi:10.3389/frtra.2024.1404740)
Supplement: Supplementary file 1 [file Datasheet1.pdf]

Propionic acid supplementation promotes the expansion of regulatory T cells in patients with end-stage renal disease but not in renal transplant patients

Moritz Anft[1]†, Fabian Meyer[1,2]†, Sirin Czygan[3], Felix Sebastian Seibert[1], Benjamin Johannes Rohn[1], Fotios Tsimas[1,3], Richard Viebahn[3], Timm Westhoff[1], Ulrik Stervbo[1], Nina Babel[1,4]*, Panagiota Zgoura[5]

†These authors contributed equally to this work and share first authorship

[1]Center for Translational Medicine and Immune Diagnostics Laboratory, Medical Department I, Marien Hospital Herne, University Hospital of the Ruhr-University Bochum, Hölkeskampring 40, 44625 Herne, Germany

[2]Dept. Anestesiology, Knappschaftskrankenhaus Bochum, In der Schornau 23-25, 44892 Bochum, Germany

[3]Dept. Surgery, Knappschaftskrankenhaus Bochum, In der Schornau 23-25, 44892 Bochum, Germany

[4]Berlin Institute of Health, Berlin-Brandenburg Center for Regenerative Therapies, and Institute of Medical Immunology, Charité – Universitätsmedizin Berlin, Corporate Member of Freie Universität Berlin, Humboldt-Universität zu Berlin Augustenburger Platz 1, 13353 Berlin, Germany

[5]Clinic for internal medicine, St. Anna Hospital Herne, Hospitalstraße 19, 44649 Herne, Germany

Supplemental Table S1

Antigen	Fluorophore	Clone	Manufacturer	Dilution
**Beta7**	PerCP/Cy5.5	FIB27	Biolegend	1:100
**CCR9**	BV421	L053E8	Biolegend	1:100
**CD127**	BV650	019D5	Biolegend	1:100
**CD137**	PE-Cy7	4B4-1	Biolegend	1:100
**CD14**	PE-Vio770	TÜK4	Miltenyi Biotec	1:100
**CD154 (CD40L)**	AF647	24-31	Biolegend	1:100
**CD16**	APC-Vio770	EA423	Miltenyi Biotec	1:100
**CD19**	BV605	HIB19	Biolegend	1:200
**CD25**	PE-Cy7	2A3	BD Bioscience	1:25
**CD3**	BV785	OKT3.	Biolegend	1:200
**CD4**	AF700	OKT4	Biolegend	1:50
**CD45RA**	BV605	HI100	Biolegend	1:200
**CD56**	PerCP-Cy5.5	NCAM	Biolegend	1:50
**CD8**	V500	RPA-T8	BD Bioscience	1:50
**FoxP3**	PE	PCH101	Thermo Fisher Scientific	1:10
**Helios**	AF647	22F6	Biolegend	1:25
**HLA-DR**	BV650	L243	Biolegend	1:100
**CCR7**	AF488	G043H7	Biolegend	1:50
**CD45**	AF488	2D1	Biolegend	1:200
**Fixable Live/Dead**			eBioscience	1:1000

AF = Alexa Fluor; BV = Brilliant Violett

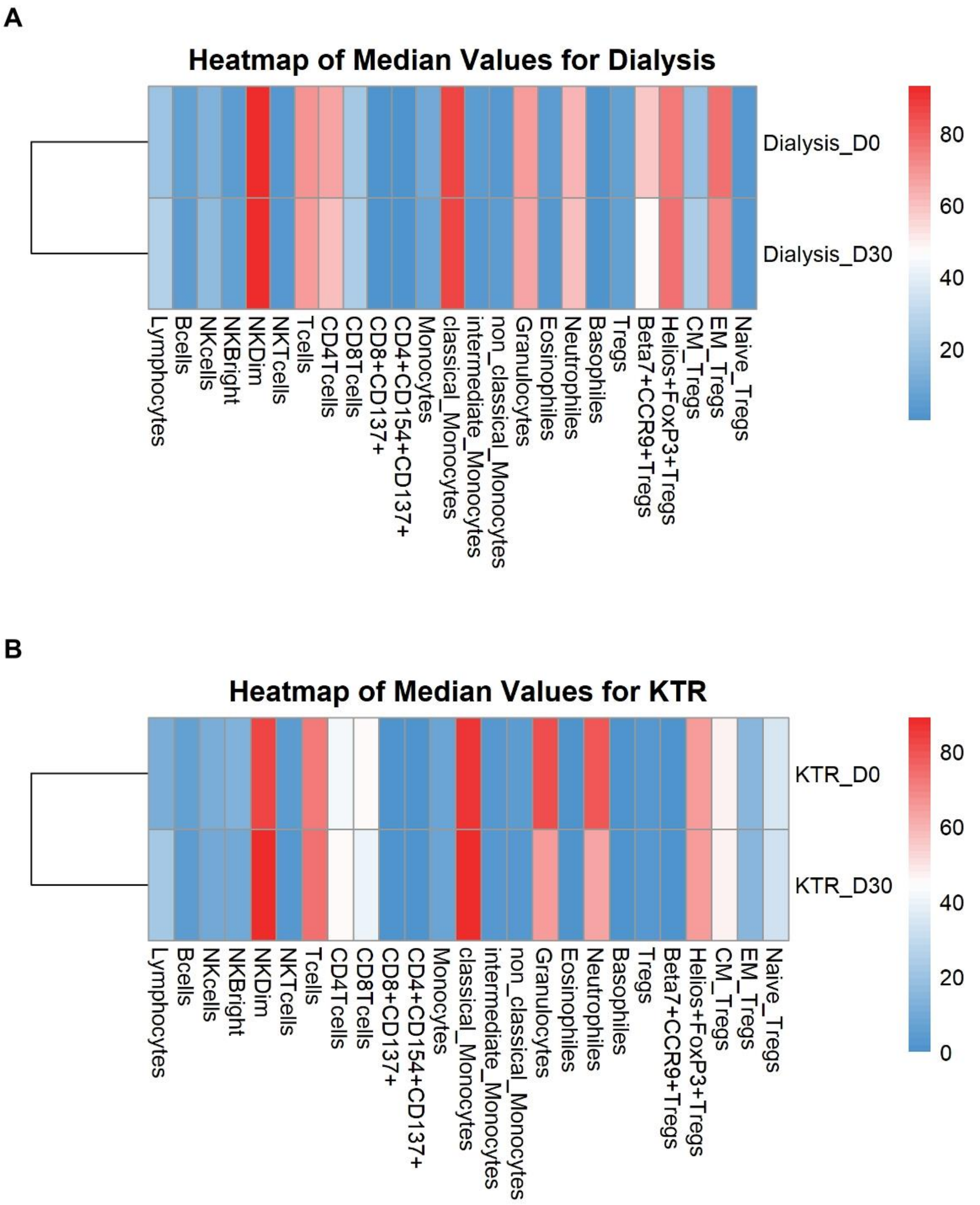

Supplemental Figure S1: Heatmap of immune cell populations before and after propionate supplementation. Heatmap with median percentage values of the immune cell populations of hemodialysis patients (A) and KTR (B) before (D0) and after (D30) propionate supplementation.

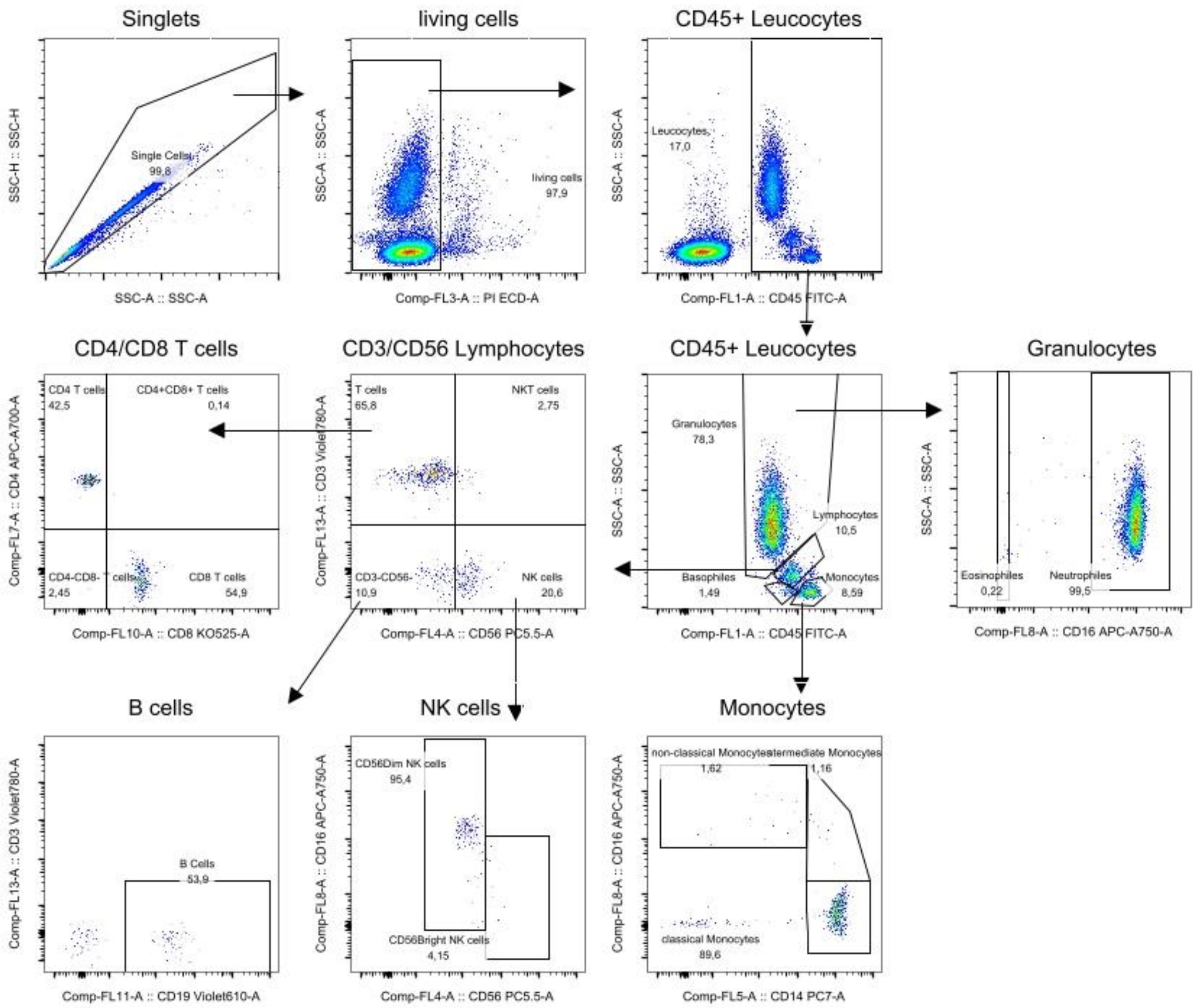

Supplemental Figure S2. Gating strategy for basic leucocyte populations. Whole blood was stained with antibody panel for basic immune cells. Leucocytes were identified by CD45 and Lymphocytes, Monocytes, Granulocytes and Basophils by characteristic CD45/SSC-profile. B cells were identified as CD3-CD19+ Lymphocytes and NKT cells as CD3+CD56+ Lymphocytes. NK cells were identified as CD3-CD56+ and further distinguished in CD16+CD56Dim NK cells and CD16-CD56Bright NK cells. Monocytes were grouped by expression of CD14 and CD16 in CD14+CD16- classical Monocytes, CD14+CD16+ intermediate Monocytes and CD14dimCD16+ non-classical Monocytes. Granulocytes were distinguished in CD16+ neutrophiles and CD16- eosinophiles.

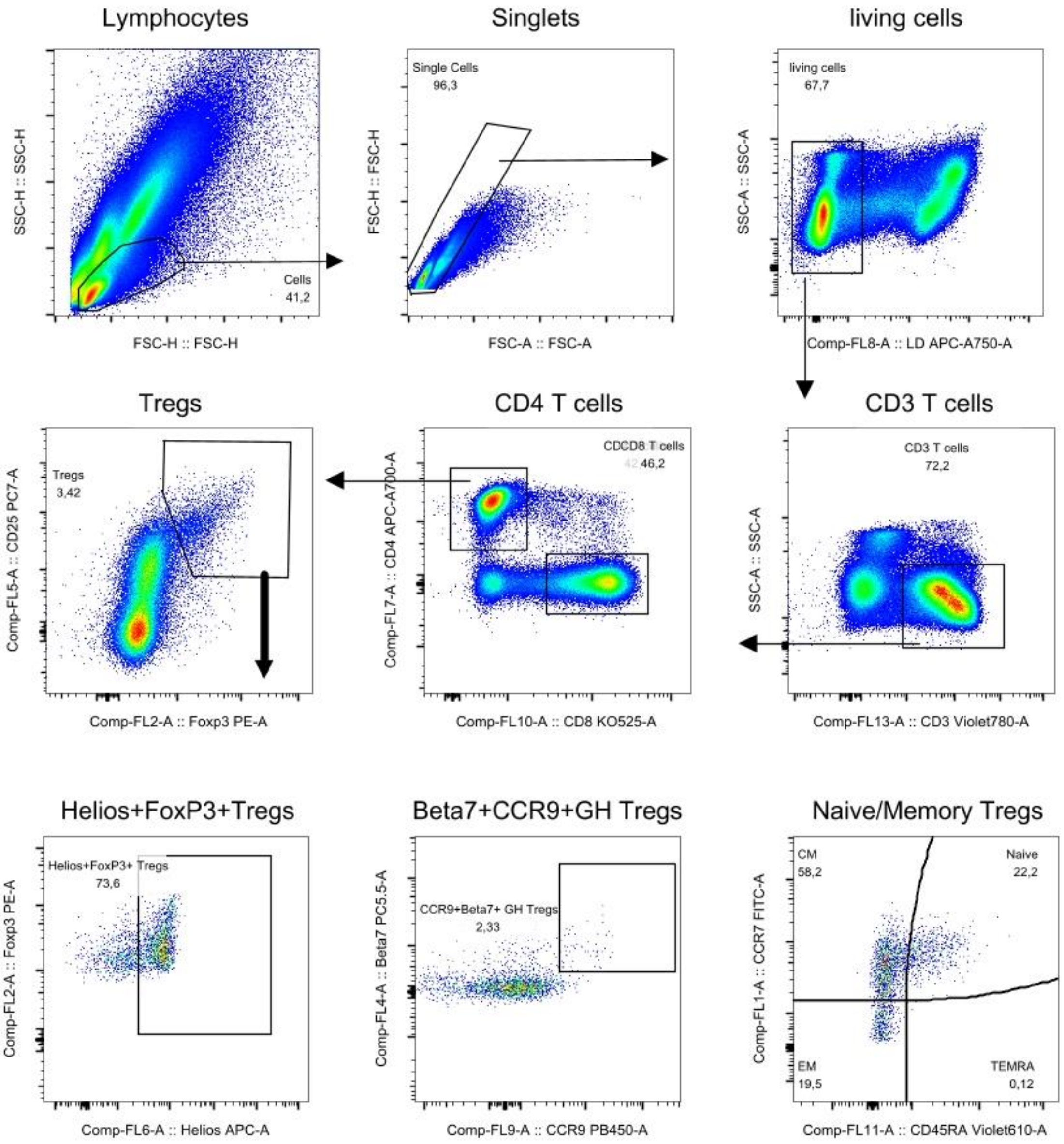

Supplemental Figure S3. Gating strategy for GH Tregs. Lymphocytes were identified by characteristic SSC/FSC profile. Doublets and dead cells were excluded and Tregs were identified as CD3+CD4+CD25+FoxP3+. Tregs were further characterized by expression of Helios and FoxP3. Gut homing Tregs were identified by expression of Beta7 and CCR9 and Memory Tregs were identified as naïve (CCR7+CD45RA+), central memory (CM, CCR7+CD45RA-) effector memory (EM, CCR7-CD45RA-) and Treg EMRA (CCR7-CD45RA+) cells.

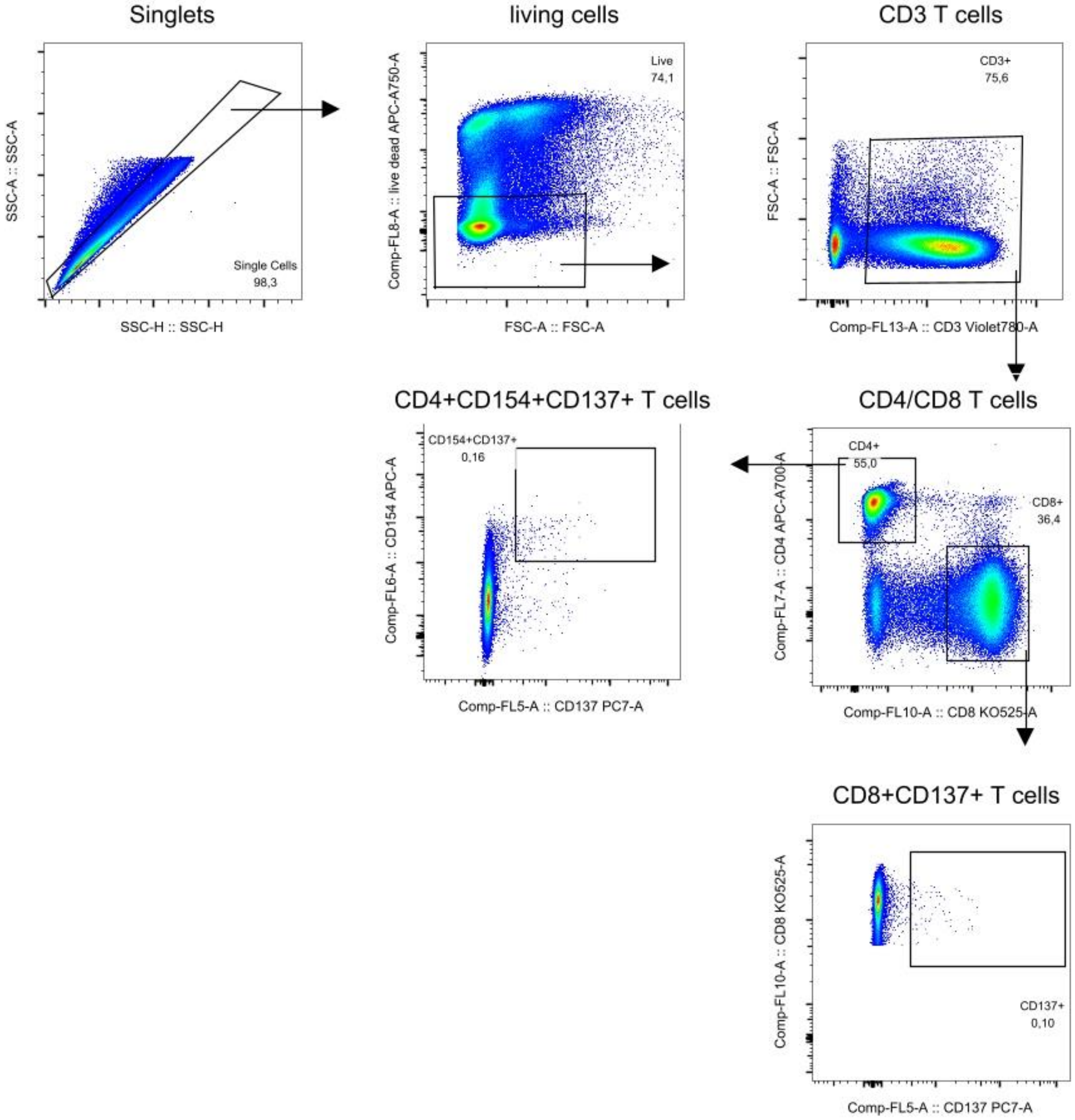

Supplemental Figure S4. Gating strategy for TD specific T cells. Isolated PBMC were stimulated with TD vaccine for 16 hours and doublets and dead cells were excluded. T cells were identified by CD3 expression and differentiated into TD-reactive CD4+CD154+CD137+ T helper cells and TD-reactive CD8+CD137+ cytotoxic T cells.
